# Supplementary material for: Prediction Accuracy of Serial Lung Ultrasound in COVID-19 Hospitalized Patients (Pred-Echovid Study)
Source: J Clin Med. 2021 Oct 20;10(21):4818. doi: 10.3390/jcm10214818 (PMC8584928; doi:10.3390/jcm10214818)

Table S1. Number of patients included in each research center and ultrasound machine used.

Hospital	Numer of patients included	Ultrasound machine
**Infanta Leonor UH**	151	Butterfly inc, / Esaote Mylab gamma
**Infanta Cristina UH**	134	Esaote Mylab alpha
**Doce de Octubre UH**	103	Phillips Lumify
**La Paz UH**	34	GE Venue ultrasound system
**Virgen del Rocio UH**	21	Butterfly inc
**Isabel Zendal**	14	GE Venue ultrasound system
**Clinico San Carlos UH**	7	Butterfly inc

Figure S1: Lung ultrasound score images.

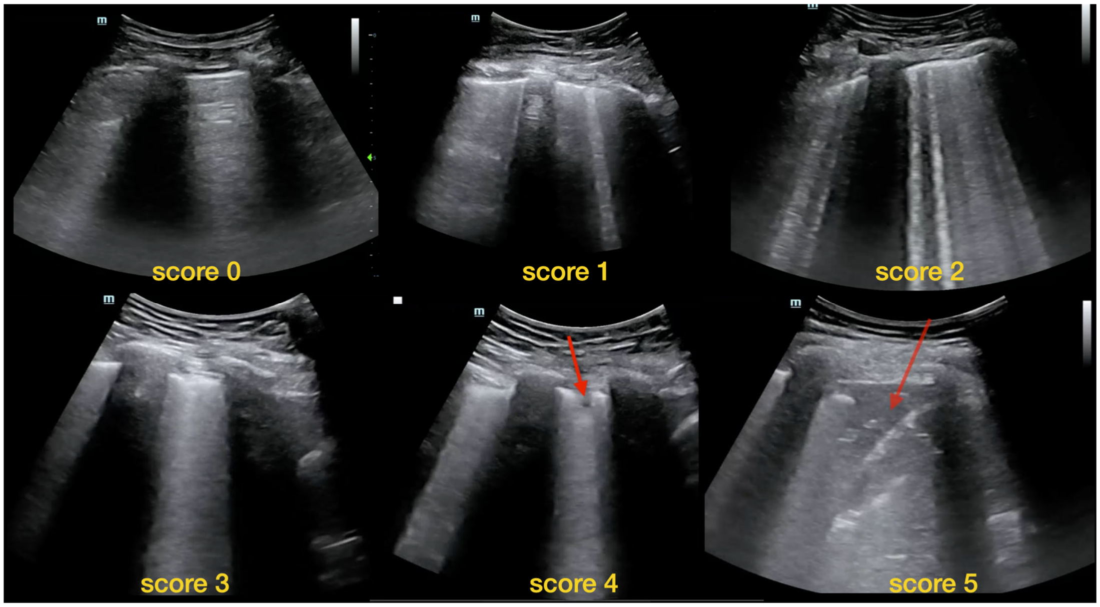

Figure S2. Calibration curve of predicted main end-point (orotracheal intubation or death) risk by SCORE-1 (point-of-care ultrasonographic score obtained during the first 24 hours of hospital stay) in a multicenter cohort of in-hospital patients with COVID-19 from Spain.

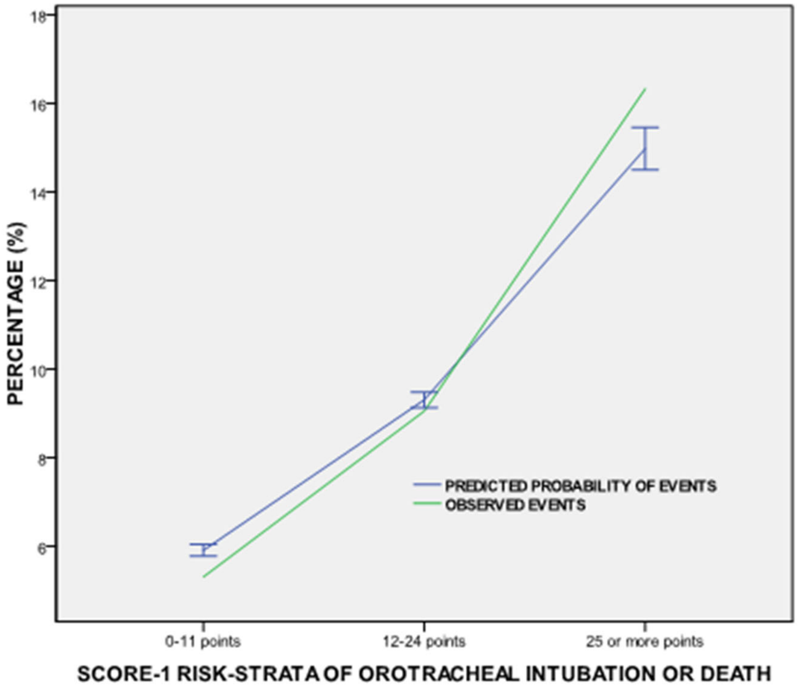

Figure S3. Calibration curve of predicted main end-point (orotracheal intubation or death) risk by SCORE-2 (point-of-care ultrasonographic score obtained after 72 hours and before 96 hours of hospital stay) in a multicenter cohort of in-hospital patients with COVID-19 from Spain.

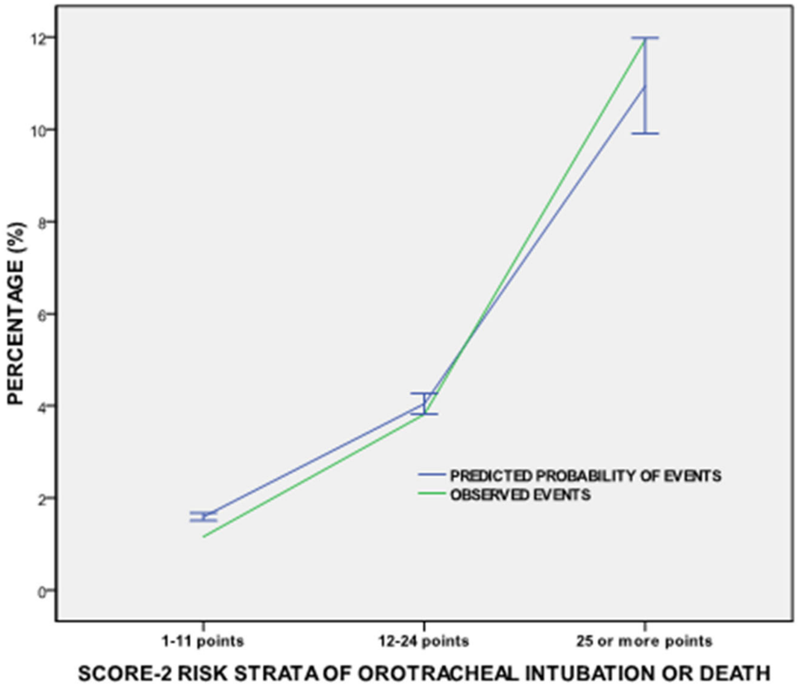

Figure S4. Calibration curve of predicted main end-point (orotracheal intubation or death) risk by DIFFERENTIAL SCORE (difference between SCORE-2 and SCORE-1) in a multicenter cohort of in-hospital patients with COVID-19 from Spain.

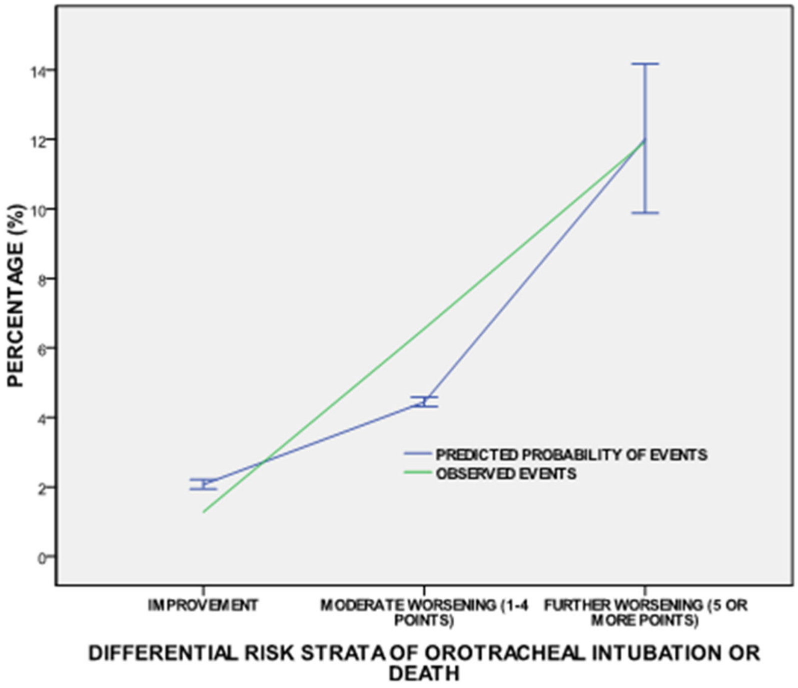

Supplement: Supplementary file 1 [file jcm-10-04818-s001.zip › jcm-1398499-supplementary.pdf]
